# The chemotherapeutic agent doxorubicin induces brain senescence, with modulation by APOE genotype

**DOI:** 10.1016/j.expneurol.2023.114609

**Published:** 2023-11-07

**Authors:** Tamar Demby, Phillip S. Gross, Jeanne Mandelblatt, Jeffrey K. Huang, G. William Rebeck

**Affiliations:** a National Institute of Diabetes and Digestive and Kidney Disease, Bethesda, MD, United States of America; b Interdisciplinary Program in Neuroscience, Georgetown University, Washington, DC, United States of America; c Department of Oncology, Georgetown Lombardi Comprehensive Cancer Center and Georgetown Lombardi Institute for Cancer and Aging Research, Georgetown University, Washington, DC, United States of America; d Department of Biology, Georgetown University, Washington, DC, United States of America; e Department of Neuroscience, Georgetown University, Washington, DC, United States of America

**Keywords:** ApoE, Apolipoprotein E, Alzheimer’s disease, Cancer chemotherapy-induced cognitive, impairment, Senescence

## Abstract

Many cancer patients experience serious cognitive problems related to their treatment, which can greatly affect their quality of life. The molecular mechanisms of this cancer chemotherapy-induced cognitive impairment (CICI) are unknown, thus slowing the development of preventative approaches. We hypothesized that cancer chemotherapies could induce cellular senescence in the brain, creating a pro-inflammatory environment and damaging normal brain communication. We tested this hypothesis using the common chemotherapeutic agent doxorubicin in two independent mouse models. In the first model, we used mice that express tdTomato under the *pdkn2a* (p16) promoter; p16 is a regulator of cellular senescence, and its upregulation is denoted by the presence of fluorescently tagged cells. Two weeks after exposure to three doses of 5 mg/kg doxorubicin, the number of tdTomato positive cells were increased nearly three-fold in both the cerebral cortex and the hippocampus. tdTomato staining co-localized with neurons, microglia, oligodendrocyte precursor cells, and endothelial cells, but not astrocytes. In the second model, we used *APOE* knock-in mice, since the *APOE4* allele is a risk factor for CICI in humans and mouse models. We isolated RNA from the cerebral cortex of *APOE3* and *APOE4* mice from one to 21 days after a single dose of 10 mg/kg doxorubicin. Using NanoString analysis of over 700 genes related to neuroinflammation and RT-qPCR analysis of cerebral cortex transcripts, we found two-fold induction of four senescence-related genes at three weeks in the *APOE4* mice compared to the *APOE3* control mice: p21(*cdkn1a*), p16, *Gadd45a*, and *Egr1*. We conclude that doxorubicin promotes cellular senescence pathways in the brain, supporting the hypothesis that drugs to eliminate senescent cells could be useful in preventing CICI.

## 1. Introduction

A debilitating side effect of cancer chemotherapy is acute and chronic forms of cognitive impairment, which can include problems with learning and memory, attention, and processing speed (Carroll et al., 2022). Pre-clinical and clinical studies have led to various hypotheses about the mechanisms of this cancer chemotherapy-induced cognitive impairment (CICI) (Fernandez et al., 2020), including oxidative damage to a variety of macromolecules (Alhowail et al., 2019; Konat et al., 2008; Rummel et al., 2021), inhibition of neurogenesis due to disruption of DNA replication (Dietrich et al., 2006; Usmani et al., 2023; Yang et al., 2010), induction of peripheral inflammation and subsequent infiltration of immune cells into the CNS (Ren et al., 2017; Shi et al., 2019; Williams et al., 2018), and disruption of the blood brain barrier leading to brain inflammation (Branca et al., 2018). The mechanisms investigated in the mouse models all represent various aspects of aging processes, suggesting that common chemotherapeutic agents are drivers of aging (Cavalier et al., 2021).

One aging mechanism that is consistent with observed chronic cognitive impairment is the induction of CNS cellular senescence (Campisi, 2013; Demaria et al., 2017). Senescence is a process by which damaged cells (including glial cells in the CNS) no longer pass through the cell cycle, but remain metabolically active and continue to perform some of their functions (Baker et al., 2023). In particular, affected microglia increase secretion of a number of senescence-associated secretory proteins, such as IL-1β, TGF-β, and matrix metalloproteases, that contribute to chronic inflammation (Lucas et al., 2023). In fact, senescent glia occur in a number of contexts, including Alzheimer’s disease (AD) (Lau et al., 2023) and normal aging (Campisi et al., 2019).

Insight into the biological mechanisms of CICI has been provided from human genetics. Several studies have demonstrated that the ε4 allele of the Apolipoprotein E (*APOE*) gene is a risk factor for CICI (Ahles et al., 2003; Ahles et al., 2014; Amidi et al., 2017; Koleck et al., 2014; Mandelblatt et al., 2018). Human *APOE* exists as three common alleles (*APOE2*, *APOE3*, and *APOE4*); *APOE4* occurs in nearly 25% of the US population, and is the strongest genetic risk factor for dementia associated with neurodegenerative diseases such as AD and Diffuse Lewy Body Disease (Chen et al., 2021; Dickson et al., 2018; Zhao et al., 2020). Individuals carrying *APOE4* showed significantly more CICI in treatments of breast cancer (Ahles et al., 2003; Mandelblatt et al., 2018) and testicular cancer (Amidi et al., 2017) than those lacking *APOE4*. The susceptibility of *APOE4*-positive cancer survivors to CICI is supported by preclinical studies: compared to *APOE3, APOE4* knock-in mice have an increased sensitivity to chemotherapy-induced behavioral impairment in spatial learning at 6 months of age (Speidell et al., 2019) and 12 months of age (Demby et al., 2020). These data support a role of inflammation in CICI, since *APOE* encodes a lipid transport protein that modulates inflammation in the periphery and the CNS (Kloske and Wilcock, 2020; Parhizkar and Holtzman, 2022).

In the current study, we use two mouse models to test different mechanisms related to aging processes and genetic risk for neuro-degeneration after exposure to doxorubicin, a common cancer therapeutic agent. One model was developed specifically to test for the induction of the senescence related gene *Cdnk2a* (p16), and one model was chosen to examine any effects that were related to *APOE* genotype. We found that doxorubicin increases senescence in the cerebral cortex and hippocampus, and that induction of senescence-related genes is increased by the presence of *APOE4*. These results may provide targets for treatments to prevent or ameliorate CICI.

## 2. Materials and methods

### 2.1. Mice

All animal experiments, procedures, and handling were performed according to protocols approved by the Georgetown University Institutional Animal Care and Use Committee, following all ethical standards and guidelines for animal welfare, including the National Institutes of Health Guide for the Care and Use of Laboratory Animals.

In order to measure the induction of the *Cdkn2a* (p16) promoter in vivo, we used B6J.Cg-*Cdkn2atm4Nesh*/Mmnc mice (NIH Mutant Mouse Resource & Research Center). The transgene uses the endogenous *Cdkn2a* promoter to drive expression of a tdTomato construct (Liu et al., 2019). These mice are heterozygous for the p16-tdTomato allele, expressing red fluorescence when the promoter is activated. We used six-month old mice (both male and female, *n* = 3–4 mice per treatment group) to reflect effects of our chemotherapeutic agent, doxorubicin, on any cancer where the drug is used.

For experiments on the effect of *APOE* genotype on the response of mice to chemotherapy, we used six-month old female *APOE* targeted-replacement mice on a C57BL/6 J background (*APOE*-TR (Sullivan et al., 1997)). These mice express *APOE3* or *APOE4* under the endogenous mouse *Apoe* promoter (rather than the mouse *Apoe*) (Sullivan et al., 1997). They allow exploration of the effects of the *APOE4* allele risk on normal brain function or in the presence of various stressors compared to the most common *APOE3* allele (Lewandowski et al., 2020). Female *APOE3* (*n* = 36) and *APOE4* (*n* = 32) mice were used for these experiments because previous clinical (Mandelblatt et al., 2018) and preclinical (Demby et al., 2020; Speidell et al., 2019) work related to *APOE* focused on treatment of breast cancer, which is observed primarily in women.

### 2.2. Chemotherapeutic drug exposure

**Fig. 1.**
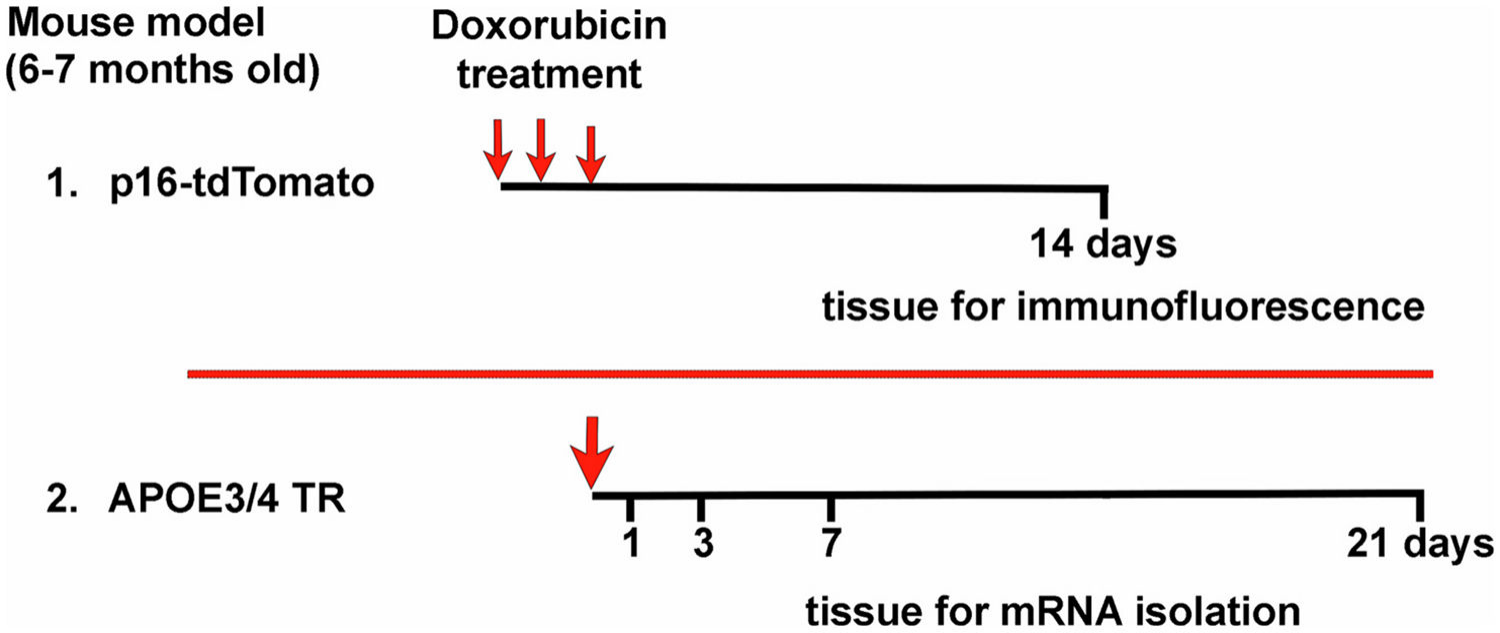
Doxorubicin treatment paradigms for the two mouse models. 1. Mice with a tdTomato construct driven by the endogenous p16 (*Cdkn2a*) promoter (*n* = 3–4) were injected at six-months of age with saline or doxorubicin, at 5 mg/kg daily over three days (small red arrows). Mice were euthanized 14 days later, and brain tissue was collected for immunofluorescence. 2. *APOE* targeted replacement (*APOE TR*) mice at 6.5 to 7.5 months of age (n = 6–8 per group) were injected once with saline or doxorubicin, at 10 mg/kg (large red arrow). Mice were euthanized from 1 to 21 days later and brain tissue collected for RNA isolation and analysis.

Doxorubicin hydrochloride was prepared as a 10 mM solution in ultrapure DMSO and kept at 4 °C for a maximum of one month before use. Doxorubicin was diluted in sterile phosphate-buffered saline (PBS) and administered via intraperitoneal injection. For p16-tdTomato experiments, mice were injected with doxorubicin at 5 mg/kg or PBS vehicle control for three consecutive days. Two weeks after the final treatment, mice were euthanized by isoflurane and cardiac perfused with 15 ml ice cold PBS (Fig. 1). Brains were extracted and fixed in 4% paraformaldehyde in PBS for 6 h followed by sucrose gradient and embedding in optimal cutting temperature (OCT) compound.

For *APOE*-TR mouse experiments, mice were injected once with vehicle control or 10 mg/kg doxorubicin; a single treatment was chosen to allow a simple determination of changes to gene expression from the time of exposure. Mice exposed to doxorubicin were euthanized one, three, seven, or 21 days following the injection by CO_2_ inhalation; control mice exposed to PBS were euthanized three days following injection (*n* = 6–8 mice per treatment condition, *APOE* genotype and time point) (Fig. 1). The mice were perfused with 5–10 ml of ice-cold PBS, and perfused brains were hemisected, with one hemisphere dissected into cortex, cerebellum, and hippocampus, and snap-frozen.

### 2.3. Immunohistochemistry

Fixed, OCT-embedded brains were sectioned coronally at 12um on a cryostat and stored at −80 °C. Slides were dried for 1 h followed by antigen retrieval according to manufacturer’s instructions (Vector Laboratories). Sections were washed and permeabilized before being blocked with 5% donkey serum, 1% bovine serum albumin, and 0.4% Triton-X in Tris-buffered saline. Primary antibodies were diluted in blocking buffer and applied overnight, followed by fluorophore-conjugated secondary antibodies and DAPI (the primary and secondary antibodies used are shown in Table 1). The tdTomato protein was visualized using an anti-RFP antibody raised in rabbit, and cell-type specific proteins were visualized with antibodies raised in other species. Slides were then mounted with fluoromount and coverslips, and allowed to dry. Images were collected on a Zeiss LSM 800 complete system confocal imager. Regions analyzed were the cerebral cortex and hippocampus (dentate gyrus and hilus), as well as the hypothalamus and choroid plexus. Two sections per region per brain were imaged. Confocal files were analyzed in ImageJ, where background was subtracted from individual channel tiffs. For each channel, tiffs were thresholded to a predetermined value and the percentage of the area field of view was measured. For co-localization, thresholded tiffs were processed through image calculator “AND” function before the percent area was measured.

### 2.4. RNA analyses

Frozen samples of cerebral cortex (30–60 mg by weight) were used for RNA isolation using the Direct-zol RNA Miniprep Kit according to manufacturer’s instructions (Zymo Research). RNA quantity and quality were confirmed using Nanodrop spectroscopy. The nCounter^®^ Mouse Neuroinflammation Panel was used to quantify the absolute number of transcripts for 770 neuroinflammation-related genes (Supplemental data); it was performed at the Genomics & Epigenomics Shared Resource at Georgetown University. RNA was pooled from four brains for each of eight groups: *APOE3* control, and 3-, 7-, and 21-days post-doxorubicin exposure; and *APOE4* control, and 3-, 7-, and 21-days postdoxorubicin exposure. Data normalization for absolute transcript counts was based on housekeeping genes included in the panel. Genes of interest were identified based on fold-change differences of ≥1.5 between control and one or more doxorubicin-exposed time points in either or both *APOE3* and *APOE4* genotypes.

For cDNA preparation from isolated RNA samples, the High Capacity cDNA Reverse Transcription Kit (Applied Biosystems) was used with 600–1000 ng total RNA; cDNA synthesis was performed according to manufacturer’s instructions. For quantitative analysis of transcripts, RT-qPCR was performed using cDNA samples diluted either 1:25 or 1:30 and normalized to GAPDH for each plate run. Samples were run in triplicate (*n* = 4–8 brains per group). Primers used for transcript analysis are shown in Table 2.

### 2.5. Statistical analyses

For analysis of tdTomato positive cell numbers, Student’s *t*-tests were used to compare doxorubicin-treated and untreated brains. For Nano-String nCounter^®^ Mouse Neuroinflammation Panel, data normalization generated transcript counts for all panel genes, based on housekeeping genes included in the panel for this purpose. For initial analysis of RT-qPCR time course data, GraphPad Prism 8 was used to perform two-way ANOVA with Tukey’s multiple comparisons tests. Statistical significance was determined with *p* < 0.05). For the subsequent comparison of RT-qPCR of controls with day 21 doxorubicin samples, outliers were identified and removed using the ROUT method (Q = 0.1%). Those data were then assessed using two-way ANOVA.

## 3. Results

**Fig. 2.**
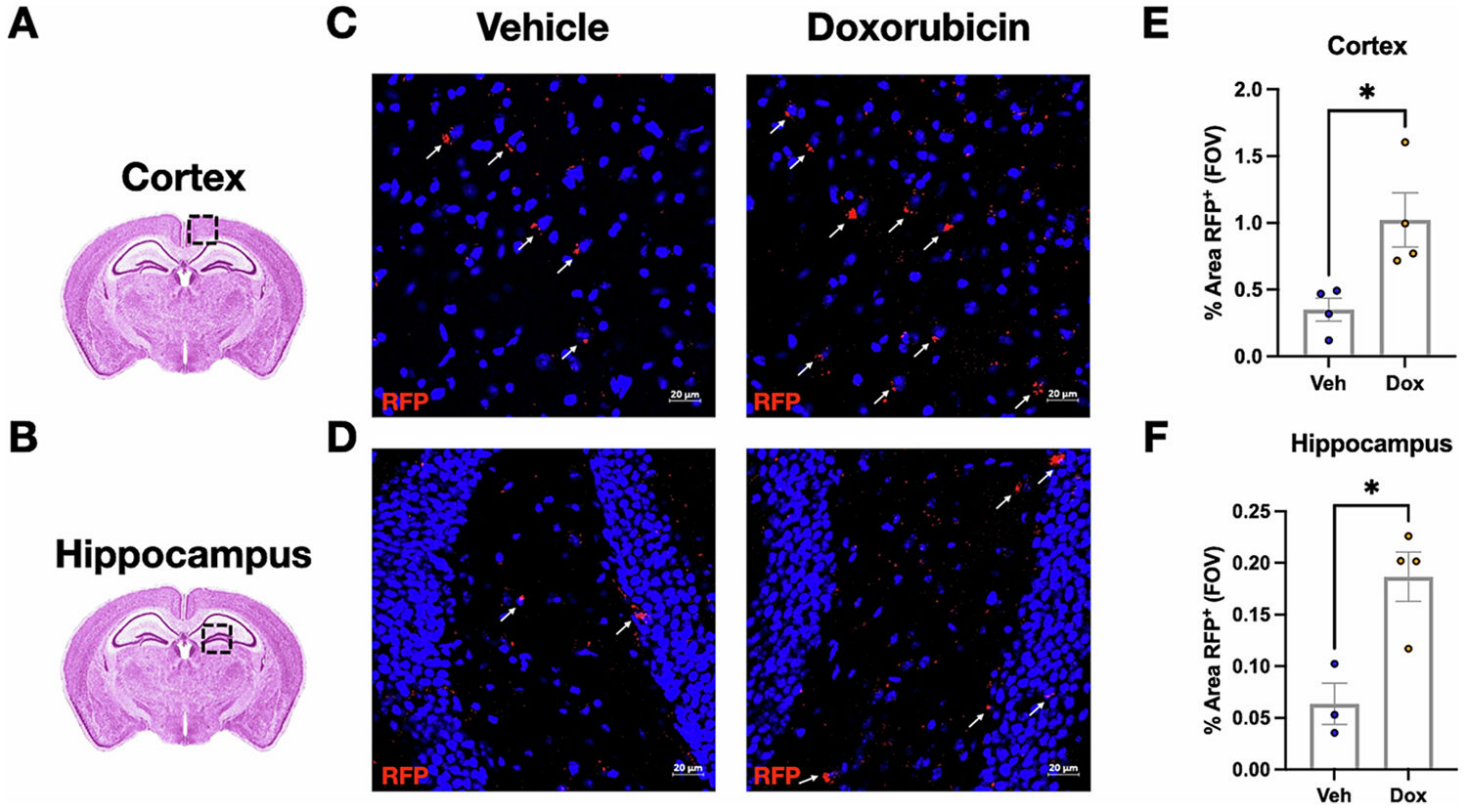
Doxorubicin treatment increases p16-tdtomato expression. (A) Cerebral cortex and (B) hippocampus were analyzed for tdTomato expression. Mice were treated with vehicle or doxorubicin, and immunofluorescent staining was used to measure p16-tdtomato expression in the cortex (C) and hippocampus (D); the red areas, indicated by white arrows, demonstrate cells expressing tdTomato from the p16 promoter. The percentage of area covered by red staining in the field of view (FOV) was measured (E and F), and statistically compared between mice exposed to vehicle and mice exposed to doxorubicin (*n* = 3–4/group, Student’s T-test, *p < 0.05).

We tested if peripheral doxorubicin treatment of mice increased brain expression of senescence markers using the p16-tdTomato mice, which express a tdTomato fluorophore expressed under the endogenous *p16INK4A* promotor (Liu et al., 2019). Fourteen days after three doses of doxorubicin or PBS control (Fig. 1), the presence of tdTomato-positive cells in the brain was examined by immunostaining analysis. We examined the cerebral cortex and hippocampus (Fig 2A and B) as well as hypothalamus and choroid plexus. The levels of p16 expression were measured by the percent area of the brain regions covered by tdTomato staining. We observed sparse tdTomato positive cells in the cerebral cortex (Fig. 2C) and in the hippocampus (Fig. 2D) in the control mice; occasional tdTomato positive cells were also observed in the hypothalamus and choroid plexus (data not shown). Comparing across treatment conditions, we found a strong and statistically significant increase in p16-tdtomato positivity in the cortex (2.9 fold) and hippocampus (2.9 fold) in doxorubicin-treated mice compared to controls (*p* < 0.05; Fig. 2E and F).

**Fig. 3.**
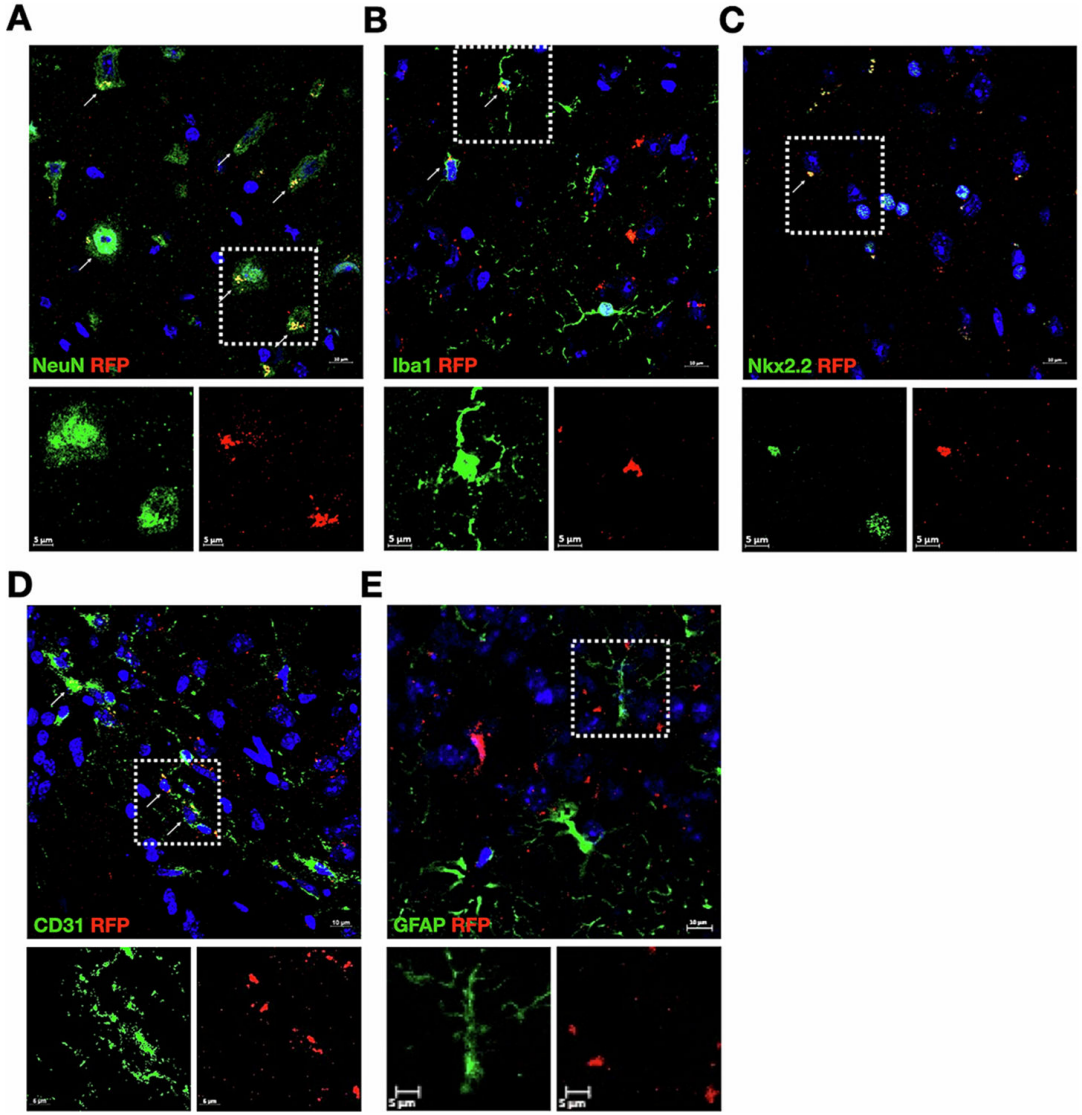
Doxorubicin-treated mice show p16 expression in several CNS cell types. Tissue from the experiment in Fig. 2 was co-stained for tdTomato (in red, for p16 expression) and antibodies to specific CNS cell types (in green). (A) NeuN for neurons; (B) Iba1 for microglia; (C) Nkx2.2 for OPC; (D) CD31 for endothelia; (E) GFAP for astrocytes. In the upper, larger panels, green staining indicates components of specific cell types, red staining indicates p16 expression not colocalizing with the cell type, and yellow indicates overlap of the cell type with p16 expression (indicated by white arrows). The areas indicated by white dotted squares are enlarged in the two panels below, showing the cell types (left, in green) and the p16 expression (in red) independently. Colocalization of p16 expression is seen in the images for neurons (A), microglia (B), OPCs (C), and endothelial cells (D), but not astrocytes (E).

We next determined which cells in the brains of these mice expressed p16-tdTomato after doxorubicin treatment by co-staining for cell specific markers: neurons – NeuN; microglia - Iba1; oligodendrocyte precursor cells (OPC) - Nkx2.2; Cd31/PECAM-1 – endothelial cells; astrocytes – GFAP, followed by confocal microscopy analysis. We observed the expected morphology and distributions of the cell types: NeuN stained neuronal nuclei; Iba1 stained whole microglia; Nkx2.2 stained nuclei, consistent with its function as a transcription factor; Cd31 stained cells with vascular phenotypes; GFAP stained intracellular cytoskeleton. We found that tdTomato was detected primarily in neurons (Fig. 3A) and microglia (Fig. 3B). Additional tdTomato positivity was observed in OPCs (Fig. 3C) and endothelial cells (Fig. 3D). We saw no tdTomato staining in astrocytes (Fig. 3E). Thus, p16 expression after doxorubicin exposure occurred in several different CNS cell types.

Clinical (Ahles et al., 2003; Mandelblatt et al., 2018) and preclinical (Demby et al., 2020; Speidell et al., 2019) studies show that *APOE4* genotype predisposes to CNS impairments due to cancer chemotherapy. In order to test this association with respect to senescence, we tested the effects of doxorubicin on *APOE3* and *APOE4 TR* mice. Mice were treated with doxorubicin once in order to define the time course of gene induction, isolating RNA from brain cortices at different times after doxorubicin treatment (Fig. 1). For an initial analysis of neuro-inflammatory transcripts, we used a large panel of neuroinflammation genes on RNA samples from four brains per group for *APOE3* and *APOE4* control mice and mice treated with doxorubicin. Eight genes with at least 1.5-fold differences from controls on at least one timepoint were identified using this approach: *Arc, Cdkn1a, Egr1, Eomes, fos, Sall1, Slc17a6*, and *Ttr* (Table 3). We were particularly interested in the induction of *cdkn1a*, cyclin-dependent kinase inhibitor 1 A (also known as p21), which is a cell cycle inhibitor and another major driver of cellular senescence in conditions of aging and DNA damage (Bunz et al., 1998; Jackson and Pereira-Smith, 2006; Jung et al., 2010; Macleod et al., 1995). We observed an increase in *Cdkn1a* in the pooled samples from both *APOE3* and *APOE4* mice at early time points, and the increase persisted in the *APOE4* mice at 21 days. Another gene identified, *Egr1*, is an upstream regulator of p53-mediated senescence (Krones-Herzig et al., 2003) and *Cdkn1a* mRNA expression (Ragione et al., 2003). *Cdkn2a*, which we analyzed above through the p16-tdTomato mice, was not included in the Neuroinflammation panel.

**Fig. 4.**
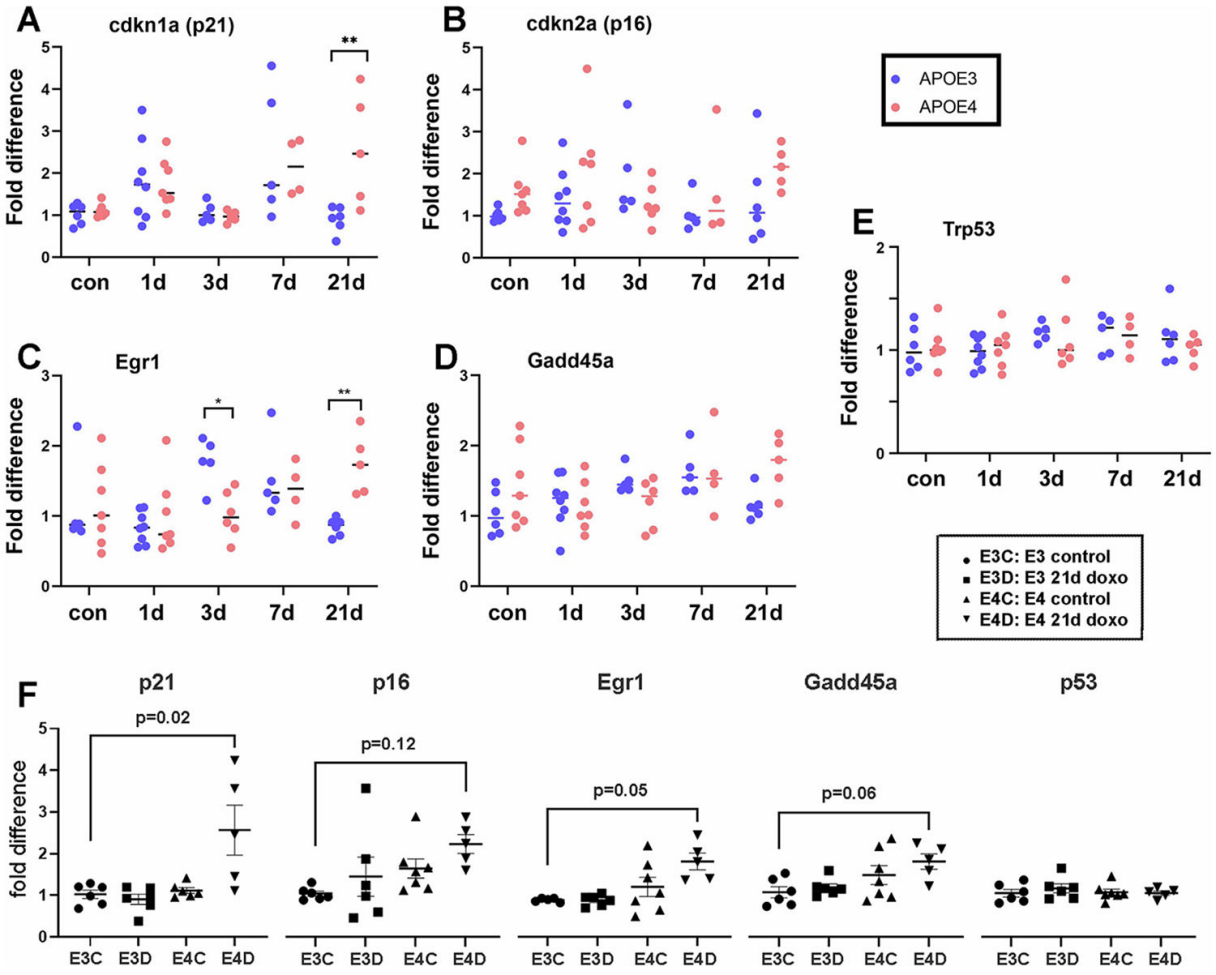
Doxorubicin induces expression of senescence-related genes. RT-qPCR was used to quantify expression of five genes after a single exposure to doxorubicin are different time points, from 3 to 21 days. The ΔΔct method was used, based in the GAPDH housekeeping gene and average Δct for *APOE3* control group for each gene; each are normalized to the levels in *APOE3* control samples. A) *Cdkn1a* ANOVA: difference by time F (4, 48) = 5.21, *p* < 0.001; difference by time*genotype interaction F (4, 48) = 2.95, *p* < 0.05; *APOE3* vs. *APOE4* 21 day, *p* < 0.01. *APOE3* 7 day vs. control, *p* < 0.05; vs. 3 day, *p* < 0.05; vs. 21 day, *p* < 0.05. *APOE4* 21 day vs. control, *p* < 0.05; vs. 3 day *p* < 0.01. B) *Cdkn2a* ANOVA: no significant group effects. C) *Egr1* ANOVA, difference by time F (4, 49) = 2.93, *p* < 0.05; interaction F (4, 49) = 4.99, *p* < 0.01; *APOE3* vs. *APOE4* 3 day difference, *p* < 0.05, 21 day, *p* < 0.01; *APOE3* control vs. 3 day, *p* < 0.01, 3 day vs. 21 day, *p* < 01; *APOE4* 1 vs. 21 day, *p* < 0.05). D) *Gadd45α* ANOVA, difference by time*genotype interaction F (4, 49) = 2.59, *p* = 0.048; *APOE3* control vs. *APOE4* 21 day, *p* = 0.028. Two-way ANOVA with Tukey’s multiple comparisons test, Šídák’s multiple comparisons test, or Dunnett’s multiple comparisons test as appropriate. E) *Trp53* ANOVA: no significant group effects. F) Direct comparisons of data from panels A-E, showing data for *APOE3* and *APOE4* control and 21 day post-doxorubicin exposure, ANOVA, F (3, 59) = 13.41, *p* < 0.0001. The *p* values for individual genes are indicated for the comparisons of *APOE3* control to *APOE4* doxorubicin treatment conditions.

Given the support in this initial assay for time-dependent effects of doxorubicin on senescence gene expression, we then tested the levels mRNA of senescence-related genes from *APOE3* and *APOE4* mice treated with doxorubicin using RT-qPCR. Both *APOE* genotypes showed increases of *Cdkn1a* after doxorubicin treatment: *APOE3* mice had significantly higher expression of *Cdkn1a* at seven days post-doxorubicin exposure compared to control; *APOE4* mice had significantly higher expression of *Cdkn1a* at 21 days post-doxorubicin exposure compared to control (*p* < 0.05; Fig. 4A). At 21 days post-doxorubicin exposure, *Cdkn1a* was also significantly higher in *APOE4* mice than *APOE3* mice (*p* < 0.01; Fig. 4A).

We used RT-qPCR to examine four other senescence related genes in the brain cortical RNA isolated from control and doxorubicin-treated mice. We tested *Cdkn2a* (which encodes p16) given the results showing its induction by doxorubicin in our other mouse model. We also tested genes based on their proximity to *Cdkn1a* expression in senescence pathways (*Cdkn2a* and *Trp53*; (Childs et al., 2015)) or their connection to senescence in cancer models (*Egr1* and *Gadd45α* (Krones-Herzig et al., 2003; Wang et al., 2021; Wang et al., 1999)). Expression of *Cdkn2a* did not show statistically significant differences across *APOE* genotypes and time, with variability between samples (Fig. 4B). As with *Cdkn1a, Egr1* was significantly higher in doxorubicin-treated *APOE4* mice than doxorubicin-treated *APOE3* mice 21 days post-exposure (*p* < 0.01; Fig. 4C). The *Gadd45α* gene encodes the Growth Arrest and DNA Damage Inducible α protein, which responds to stresses that promotes senescence (Sahu et al., 2022). Expression of *Gadd45α* varied between groups based on an interaction between *APOE* genotype and time, with a significant difference observed between *APOE3* control mice and *APOE4* mice 21 days post-doxorubicin (Fig. 4D). Finally, *Trp53*, which encodes the cell cycle arrest protein p53, showed stable expression in cortical brain tissue across *APOE* genotype and exposure times (Fig. 4E).

We organized these data to focus on the combined effects of *APOE* genotype and doxorubicin at the most chronic time point, 21 days posttreatment (Fig. 4F). For the four senescence related genes that were affected by doxorubicin, we observed similar levels of increases in the doxorubicin-treated *APOE4* compared to the control-treated *APOE3* mice: *Cdkn1a* (2.4 fold of the E3 control), *Egr1* (2.0 fold), *Cdkn2a* (2.2 fold), and *Gadd45α* (1.9 fold) (Fig. 3F), although not all the changes were statistically significant in the ANOVA of these groups in this small sample (*p* = 0.02 to *p* = 0.12). Trp53 gene expression was unaffected by doxorubicin treatment in either *APOE3* or *APOE4* mice.

## 4. Discussion

CNS damage due to cancer therapy is thought to underlie cognitive impairments in humans and behavioral deficits in mice, but the contributing mechanisms remain undefined and therefore untreated (John et al., 2021; Mounier et al., 2020). One potential mechanism of action of chemotherapies is the induction of cellular senescence, a process that increases in aging and neurodegeneration (Babapour Mofrad etal., 2020; Bussian et al., 2018; Streit et al., 2020). We used two independent mouse models, and each demonstrated induction of cell senescence genes in the brain after exposure to peripheral injections of doxorubicin. In both, doxorubicin was administered over a short period of time (three days and one day), which differs from the more chronic exposures that cancer patients receive, but allowed us to investigate initial effects acutely induced by doxorubicin. Through one model, mice with p16 promoter-mediated induction of a fluorescent marker, we observed a significant increase in labeled cells two weeks after doxorubicin treatment. In another model, *APOE TR* mice, we observed elevated levels of several senescence-related mRNA transcripts three weeks after doxorubicin treatment in the context of the *APOE4* genotype. This second model was chosen due to the increased risk of CICI in *APOE4* cancer survivors through unknown mechanisms (Fernandez et al., 2020). Together, these results demonstrate that doxorubicin induces long-term changes to senescence in the cerebral cortex, which is prominently affected in CICI (de Ruiter et al., 2023).

Senescence is a cellular condition that involves regulation of a series of genes, although whether they are induced, repressed, or unchanged can vary depending on many factors (Sahu et al., 2022). In our RT-qPCR analyses, we found multiple genes with elevated expression at three weeks post-doxorubicin exposure in *APOE4* mouse brain cortex, including *Cdkn1a* (p21) (Chen et al., 2006; Jackson and Pereira-Smith, 2006) and *Egr1* (Krones-Herzig et al., 2003), with similar trends observed in *Gadd45α* (Jackson and Pereira-Smith, 2006) and *Cdkn2a* (p16). These changes supported the more preliminary findings from a NanoString neuroinflammatory panel for induction of *Cdkn1a* and *Egr1*; an effect on *Gadd45a* was also observed (1.3 fold of the levels in the *APOE4* brains), but did not reach our cutoff criteria of 1.5 fold. The final gene, *Cdkn2a* (p16), was not part of the panel. The RT-qPCR finding with *pCdkn2a* mRNA mirrored the outcome with the p16 tdTomato mice. The p16 protein has roles in both p53-dependent and independent senescence pathways (Beausejour et al., 2003; Chen et al., 2006; Robles and Adami, 1998). Gadd45α, Egr1, and p21 are all major components of the p53-controlled DNA damage and senescence induction response pathway. Unlike these other genes, p53 is not transcriptionally upregulated in senescence, but it is preferentially recruited at the *Cdkn1a* and *Gadd45α* promoters, leading to increased transcription of them (Jackson and Pereira-Smith, 2006). p21 affects cell cycle arrest in G1 and G2 (Bates et al., 1998; Cayrol et al., 1998) and is a marker of aging and senescence (Lopez-Dominguez et al., 2021) as well as other cellular processes (Safwan-Zaiter et al., 2022). Gadd45α affects cell cycle arrest in G2/M (Wang et al., 1999) and acts as an upstream regulator of p53 via p53 protein stabilization during DNA damage (Jin et al., 2003). The transcription factor Egr1 is an upstream regulator of p53-controlled replicative senescence (Krones-Herzig et al., 2003) and also acts as a direct transcriptional regulator of *Cdkn1a* (Ragione et al., 2003; Russo et al., 1995) and *Gadd45α* (Jung et al., 2021; Thyss et al., 2005).

Senescence is observed in the context of several types of CNS damages (Campisi and Robert, 2014). The chemotherapeutic agent paclitaxel induced CNS senescence in over 10% of all brain endothelial cells (Ahire et al., 2023). Direct treatment of a microglial cell line in culture with doxorubicin caused senescence after six days (Marques et al., 2020) (although doxorubicin is excluded from the CNS by the blood brain barrier (Bigotte et al., 1982; Bigotte and Olsson, 1982)). DNA damage and its related senescence are also recognized as contributors to brain aging and cognitive decline in conditions of aging and neurodegeneration (Bhat et al., 2012; Lu et al., 2004). In this study, we observed two to three-fold inductions of senescent cells in response to doxorubicin overall in both the cortex and the hippocampus. These effects could contribute to deficits in functions of brain regions that are affected in CICI, such as executive functions, and learning and memory (Mandelblatt et al., 2018).

We observed that the p16 promoter was activated after doxorubicin treatment in many cell types throughout the cortex and hippocampus. Many of the labeled cells in the p16 tdTomato brains were neurons. However, p16 activation was observed in microglia, OPCs and endothelial cells as well, consistent with induction of senescence in these cell types under various conditions. Aged microglia exhibit pronounced dystrophy (Streit et al., 2004) and increased expression of p16 (Stojiljkovic et al., 2019) *in vivo*; their loss of replicative capacity could contribute to initiation or progression of neurodegenerative processes (Lau et al., 2023; Mosher and Wyss-Coray, 2014). Endothelial cells exhibited induction of p16 after exposure of mice to chemotherapy (Ahire et al., 2023). OPCs showed senescence in the vicinity of Aβ plaques in a mouse model of amyloid (Zhang et al., 2019). Doxorubicin exposure of mice contributes to a pro-inflammatory state in the brain (Cardoso et al., 2020; Tangpong et al., 2007), perhaps related directly to its effects on microglia, endothelial cells, and OPCs. Removal of senescent cells through senolytics could be useful in counteracting the negative effects of neurodegenerative processes or other conditions that lead to senescence in the brain (Lee et al., 2021). In fact, senolytics prevented behavioral deficits in mouse models of tauopathy (Bussian et al., 2018), amyloid (Zhang et al., 2019), and paclitaxel exposure (Ahire et al., 2023).

Some of the variability observed in gene expression in our work may represent competing processes of induction of senescence pathways compared to resolution of these pathways. For some of these senescence pathway genes, upregulation appears to occur at earlier time points in *APOE3* mice and resolve before the 21-day time point. Doxorubicin may be responsible for temporary induction of processes such as DNA damage or blood-brain barrier breakdown, which are contributors to brain aging and cognitive decline (Bhat et al., 2012; Lu et al., 2004). The observed differences in the gene induction time courses suggest that damage triggers are resolved sooner in *APOE3* mice, leading to preserved cognition, while in *APOE4* mice senescence processes are prolonged and fail to resolve during the acute time frame.

In addition to greatly increased risk of AD, *APOE4* carriers are at risk for poor neurological outcomes in other contexts, including chronic processes of Diffuse Lewy Body disease (Guerreiro et al., 2018) and tauopathy (Shi et al., 2017), and of more acute conditions like complications from COVID19 (Safdari Lord et al., 2022) or chemotherapy (Mandelblatt et al., 2018). These observations suggest that there are shared processes that are affected by *APOE4* across neurological conditions. Neuroinflammation is a response to protein accumulation and to cellular disruption, and *APOE* genotype modulates inflammatory responses (Parhizkar and Holtzman, 2022; Rodriguez et al., 2014; Stephen et al., 2019; Zhu et al., 2012). These effects of *APOE* genotype would thus be connected to the early stages of protein accumulation in chronic neurodegenerative conditions, as well as any adverse effects of neuroinflammation in acute conditions. The effects of *APOE* genotype on aspects of brain senescence may represent a mechanistic connection between the *APOE4* genotype and brain dysfunction related to neuroinflammation and accelerated brain aging (Gladyshev et al., 2021).

Several limitations of this study should be noted. First, we did not measure protein levels in analyses of p53, p21, Gadd45α, and Egr1, or phosphorylated forms of these proteins or p53, limiting our understanding of how transcriptional upregulation of these genes affects senescence induction. Second, there are differences in the paradigms for the two models studied (three days vs one day treatments, two weeks vs three weeks of progression). While these experiments differed in these experimental details, their conversion on a single pathway demonstrates the rigor of the approach. These exposure paradigms are also not consistent with long exposures seen in cancer survivors, and thus chronic exposures may lead to more cellular senescence or different patterns of senescence. Third, our *APOE* studies focused on female mice given the connections between CICI and *APOE* in breast cancer survivors (Mandelblatt et al., 2018); future studies should include male mice, given the broader use of doxorubicin in other types of cancers. Lastly, the study design leaves open the question of further damage or differences in resolution of senescence at times longer than three weeks, or that treatments at older ages might have stronger effects. Future studies in this model can utilize longer-term doxorubicin treatments over several weeks, and longer-term assessments over several months, to understand how markers of senescence differ from the more acute model here on relatively young mice, and whether this senescence triggers altered brain aging trajectories.

Broadly, cancer chemotherapy can be considered to cause an accelerated aging of the brain (Cavalier et al., 2021). We focused on the specific aging-related process of cellular senescence, using two mouse models to identify specific effects of doxorubicin on this pathway. Our findings suggest that CICI represents an effect of chemotherapy on senescence pathways, and provide evidence to support future preclinical and clinical studies of senolytics to prevent or treat CICI.

## Supplementary Material

Appendix A

## Figures and Tables

**Table 1:** Primary antibodies.

Target	Species	Dilution	Information	Secondary antibody
GFAP	Mouse	1:500	Sigma G3893	Donkey anti-Mouse, Alexa Fluor^™^ 488
Iba1	Goat	1:300	Abcam ab5076	Donkey anti-Goat, Alexa Fluor^™^ 488
NeuN	Mouse	1:200	Millipore MAB377	Donkey anti-Mouse, Alexa Fluor^™^ 488
Nkx2.2	Mouse	1:100	DSHB 74.5A5-c	Donkey anti-Mouse, Alexa Fluor^™^ 488
Cd31/pecam1	Mouse	1:200	Invitrogen 14–0311-81	Donkey anti-Mouse, Alexa Fluor^™^ 488
RFP (tdTomato)	Rabbit	1:1000	Rockland 600–401-379	Cy^™^3 AffiniPure Donkey Anti-Rabbit

**Table 2:** RT-qPCR primers.

gene	Forward primer	Reverse primer
*Cdkn1a* (p21)	CGAGAACGGTGGAACTTTGAC	CAGGGCTCAGGTAGACCTTG
*Cdkn2a* (p16)	GAACTCTTTCGGTCGTACCC	CGAATCTGCACCGTAGTTGA
*Gadd45a*	TGCGAGAACGACATCAACAT	TCCCGGCAAAAACAAATAAG
*Egr1*	GACGAGTTATCCCAGCCAA	GGCAGAGGAAGACGATGAAG
*Trp53* (p53)	GTCACAGCACATGACGGAGG	TCTTCCAGATGCTCGGGATAC
*Gapdh*	GTGTTTCCTCGTCCCGTAGA	ATTCCGTTCACACCGACCTT

**Table 3:** Genes identified using nCounter® Mouse Neuroinflammation Panel.

	E3: 3d doxo vs. control	E3: 7d doxo vs. control	E3: 21d doxo vs. control	E4: 3d doxo vs. control	E4: 7d doxo vs. control	E4: 21 d doxo vs. control
***Arc***	1.10	1.26	**−1.50**	−1.13	−1.08	1.39
***Cdkn1a***	−1.12	**2.24**	−1.30	1.03	**1.75**	**2.28**
***Egr1***	1.27	1.09	**−1.55**	−1.16	−1.16	1.27
***Eomes***	1.08	**22.92**	**1.75**	−1.00	**2.42**	**9.17**
***fos***	−1.27	1.09	**−1.66**	−1.05	−1.20	1.34
***Sall1***	−1.14	**1.90**	1.02	1.13	−1.00	1.26
***Slc17a6***	**1.35**	**2.02**	1.10	−1.24	−1.17	**−1.96**
***Ttr***	**3.56**	1.18	**2.31**	**7.27**	**−4.62**	**−2.15**

Genes of interest were identified based on fold-change differences of ≥1.5 between control and one or more doxorubicin-treated samples, noted in the boxes with orange backgrounds.

## Data Availability

Data will be made available on request.

## Abbreviations:

AD: Alzheimer’s Disease
ANOVA: Analysis of variance
APOE: Apolipoprotein E
APOE TR: APOE Targeted Replacement mice
CICI: Cancer chemotherapy induced cognitive impairment
GFAP: Glial Fibrillary Associated Protein (astrocyte marker)
Iba1: Ionized calcium-binding adaptor molecule 1 (microglia marker)
NeuN: Neuronal Nuclei (neuron marker)
OPC: Oligodendrocyte Precursor Cell
PBS: Phosphate Buffered Saline
PECAM-1: Platelet endothelial cell adhesion molecule (Cd31, endothelial cell marker)
qRT-PCR: Quantitative Reverse Transcriptase-Polymerase Chain Reaction.
